# Insights Into Electrophysiological Metrics of Cochlear Health in Cochlear Implant Users Using a Computational Model

**DOI:** 10.1007/s10162-023-00924-z

**Published:** 2024-01-26

**Authors:** Marko Takanen, Stefan Strahl, Konrad Schwarz

**Affiliations:** MED-EL Medical Electronics, Research and Development, Fürstenweg 77a, 6020 Innsbruck, Austria

**Keywords:** Auditory modeling, Cochlear implants, eCAP, Inter-phase gap, Neural survival, Electrode position

## Abstract

**Purpose:**

The hearing outcomes of cochlear implant users depend on the functional status of the electrode-neuron interface inside the cochlea. This can be assessed by measuring electrically evoked compound action potentials (eCAPs). Variations in cochlear neural health and survival are reflected in eCAP-based metrics. The difficulty in translating promising results from animal studies into clinical use has raised questions about to what degree eCAP-based metrics are influenced by non-neural factors. Here, we addressed these questions using a computational model.

**Methods:**

A 2-D computational model was designed to simulate how electrical signals from the stimulating electrode reach the auditory nerve fibers distributed along the cochlea, evoking action potentials that can be recorded as compound responses at the recording electrodes. Effects of physiologically relevant variations in neural survival and in electrode-neuron and stimulating-recording electrode distances on eCAP amplitude growth functions (AGFs) were investigated.

**Results:**

In line with existing literature, the predicted eCAP AGF slopes and the inter-phase gap (IPG) effects depended on the neural survival, but only when the IPG effect was calculated as the difference between the slopes of the two AGFs expressed in linear input–output scale. As expected, shallower eCAP AGF slopes were obtained for increased stimulating-recording electrode distance and larger eCAP thresholds for greater electrode-neuron distance. These non-neural factors had also minor interference on the predicted IPG effect.

**Conclusions:**

The model predictions demonstrate previously found dependencies of eCAP metrics on neural survival and non-neural aspects. The present findings confirm data from animal studies and provide insights into applying described metrics in clinical practice.

## Introduction

Cochlear implants (CIs) restore hearing for profoundly deaf people by bypassing the degenerated hearing mechanisms and stimulating auditory nerve fibers (ANFs) directly with electrical pulses. Typically, symmetric charge-balanced biphasic pulses are used to excite the neurons to ensure that no potentially harmful charge is built up within the cochlea. The two phases are often separated by a short inter-phase gap (IPG). The duration of this gap is known to influence the stimulus amplitude required to excite neurons [1–3].

The efficacy of electrical stimulation depends on the status of the electrode-neuron interface inside the cochlea. This is influenced by the distance and orientation of the electrode contacts relative to the neurons, and their impedance values, as well as the survival and functionality of the neural population inside the cochlea. Both durations of deafness and etiology affect the condition of the neural population in CI users, as the peripheral parts of the ANF degenerate, soma and part of the central axon of the ANF demyelinate, and eventually the whole ANF is degenerated [4–8].

The status of the electrode-neuron interface can be assessed by measuring electrically evoked compound action potentials (eCAPs) using two-way telemetry [9–11]. Common approaches are to observe the changes in eCAP thresholds and/or the slopes of the eCAP amplitude-growth function (AGF) as either the leading polarity or the IPG is changed [12–19]. eCAP thresholds are expected to decrease and the slopes to become steeper on a linear input–output scale when either the IPG is prolonged or the leading polarity is changed from anodic to cathodic (in most animal models) or from cathodic to anodic (in human CI users; for a review, see [20]). These are referred to as the “IPG effect” and the “polarity effect,” respectively. Both effects are expected to be pronounced for healthy cochleae [3, 12, 14, 21, 22] and the IPG effect has been found to correlate with neural density within the cochlea [12, 14].

Promising results from animal studies [12, 14, 19] have motivated investigators to explore the use of metrics such as the IPG effect and the polarity effect as a tool to assess cochlear health. This may in turn facilitate the optimization of CI coding strategies, for example, by using focused stimulation to direct current toward regions with higher densities of healthy neural populations [23, 24]. Although encouraging results have been obtained, benefits from such optimization could not be generalized over the investigated study populations of CI users [15, 16, 25]. This may be related to the heterogeneity among CI users and their etiologies. The challenge of translating evidence from animal studies into measurable benefits for CI users has also raised questions about how well the eCAP-based metrics reflect aspects of cochlear health, and to what degree these metrics are influenced by non-neural factors such as the electrode-neuron distance [15, 16, 26].

Recently, Brochier et al. [27] presented a mathematical model and applied it to data from animals [12] and CI users [25]. In their analysis, the IPG effect as a measure of neural health was best computed as the offset between the overlapping portions of the eCAP AGFs expressed in logarithmic input–output scale. Other methods of computing the IPG effect were deemed to be biased by non-neural influences, which to some extent questioned the findings from studies using such IPG-effect metrics [12, 14, 19].

The aim of this study was to address some of the questions raised in previous publications [15, 26, 27] about the contributions of neural survival and non-neural factors on eCAP AGFs. To that end, a phenomenological 2-D model of an implanted cochlea was applied in simulated eCAP AGF measurements, varying IPGs, neural survival, and electrode contact-neuron distances. This computational modeling approach provides insights on dependencies of these factors, and thus helps investigators to apply eCAP-based metrics of cochlear health reliably in their clinical practice.

## Methods

The computational model and all simulations were implemented in Python programming language version 3.8 [28, 29]. The figures were generated using Python [30].

### Simplified Model of an Implanted Cochlea

A simplistic 2-D model was designed to predict the eCAP responses evoked by a given electrical stimulation. To that end, positions of the electrodes along the scala tympani were estimated based upon the computerized tomography (CT) data collected by Yoshimura et al. [31]. Using the average cochlear length of 33.9 mm [31] and an inverse of the Greenwood’s formula [32], the characteristic frequencies of the twelve electrode contacts of a Flex28 electrode array from [31] were converted into corresponding locations along the tympanic membrane. The inverse of the Greenwood’s formula was applied here instead of the revised one by Stakhovskaya et al. [33] because Yoshimura and colleagues used Greenwood’s formula to determine the electrodes’ characteristic frequencies based on µCT imaging results before presenting the averaged results in their work [31]. Each electrode contact of such a lateral wall electrode array was treated as a point source, and the orientation and size of the electrode contacts were not considered.

**Fig. 1:**
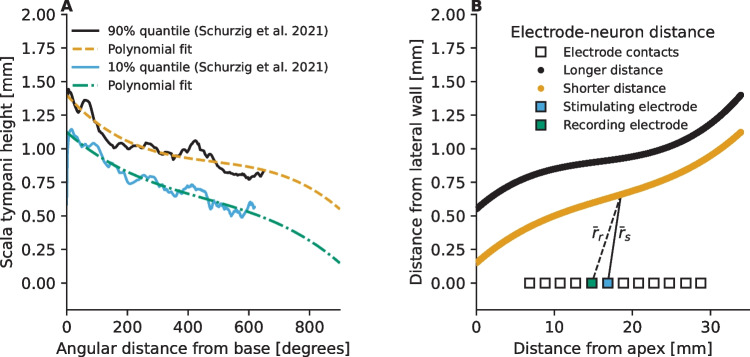
Steps taken in modeling the electrode-neuron distance in the 2-D model of an implanted cochlea. Panel **A** Polynomial equations were fitted to the 10 and 90% quantiles of the scala tympani height data from human temporal bone specimens [34]. Panel **B** Euclidean distances were computed between individual electrode contacts [31] and neurons to model the degree to which the electrical pulse emitted by the electrode contact is attenuated before reaching a given neuron, and conversely to what degree the response of an individual neuron gets attenuated before reaching the recording electrode. Here, ${\overline{r} }_{s}$ denotes the Euclidean distance vector between the stimulating electrode (electrode contact #6) and a given neuron and ${\overline{r} }_{r}$ denotes the distance vector between the same neuron and the recording electrode (electrode contact #5)

In order to estimate the distances between the electrode contacts and the neurons, data from the human temporal bone specimens reported in [34] were used. To that end, scala tympani height profiles were extracted separately for each of the 15 subjects in the dataset by calculating the average height at the middle of the scala tympani (± 0.2 mm) at the given cochlear angle. Subsequently, 10 and 90% quantiles were calculated across the individual profiles to obtain two profiles that reflect the large degree of natural variation in cochlear height profiles among individuals. Finally, third-order polynomial functions were fitted to the distributions as shown in Fig. 1A. The motivation for two separate fits was to have two electrode-neuron distance options in the simulations (Fig. 1B). It was assumed that the simulated electrode array lies on the lateral wall and that, in a healthy cochlea, there are 2000 independent, equidistantly distributed ANFs covering the entire cochlea (0 to 33.9 mm from the base, corresponding to an angular range of 0 to 900°). Additionally, we modeled conditions with 500, 1000, or 1500 independent, equidistantly distributed ANFs to simulate effects of poorer neural survival. The ANFs were assumed to share the same set of parameters. Although neural density has been found to vary along the cochlea [35], the effects investigated in this study are independent from this density variation. Therefore, the simpler uniform distribution was chosen.

Upon determining the spatial positions of the electrode contacts and the ANFs, the Euclidean distances between each electrode contact and each ANF neuron were computed and a simplified approach was applied. To simulate the spread of the electrical field, a 2 dB/mm attenuation was applied [36, 37]. The electrical current reaching a given neuron was defined as

$$\begin{array}{c}\widetilde{I}\left(t\right)= I\left(t\right)\times {10}^{\left(\frac{-2\left|{\overline{r} }_{s}\right|}{20}\right)},\end{array}$$

where $I\left(t\right)$ denotes the amplitude of the electrical signal emitted from the stimulating electrode as a function of time and ${\overline{r} }_{s}$ is the distance vector between the stimulating electrode and the given neuron (see Fig. 1B). In this study, we did not consider effects related to asymmetric spread of electrical field [38] and/or cross-turn stimulation through the bony structures [39], as such aspects would have increased the model complexity beyond the purpose of this study.

A phenomenological model [40] based on the leaky integrate-and-fire principle [41] was then used to predict the response of each individual ANF to the electrical stimulation. In this model, the ANF is assumed to integrate the incoming electrical current and to release an action potential if the membrane voltage exceeds the neuron’s stochastic threshold and if the neuron is not repolarized before it is ready to spike. The model is designed to represent one of the ANF's (peripheral or central) site of excitation that can be excited by either cathodic- or anodic-leading pulses, with the charge-balancing polarity being able to cancel the spiking by repolarizing the neuron before it is ready to spike [40]. This model is used to estimate the exact time of spiking evoked by the different pulse shapes, as well as refractoriness, accommodation and facilitation phenomena according to cat single-fiber data from literature [40, 42]. The model contains separate threshold values for anodic and cathodic polarities and the threshold values are increased temporarily after spiking to account for refractoriness and spike-rate adaptation phenomena and decreased temporarily after subthreshold stimulation to account for facilitation. The incremental effects of refractoriness and spike-rate adaptation affect the thresholds for both polarities, while the decremental effects of facilitation are polarity dependent. The exact time of spiking is predicted based on the continuously estimated spiking probability upon threshold crossing. Here, the gain coefficients of the leaky integrator (i.e., gain coefficients of the first-order infinite impulse response (IIR) filter in the model) were tripled and the standard deviation of the threshold value in the model was doubled in order to bring the eCAP threshold predictions of the overall 2-D model close to the expected 10.01 ± 3.31 nC range of eCAP thresholds among CI users [43] and to ensure monotonic behavior of spiking latency as function of stimulus amplitude, respectively. Table 1 lists the adjusted parameters of the single-fiber model.

**Table 1:** List of modified parameters of the phenomenological model of an electrically stimulated ANF [40] as they were used in this study

*Parameter*	*Description*	*Original value*	*Modified value*
*δ*	Standard deviation of the model’s threshold	4.6 µV	9.2 µV
*b*_0_ *b*_1_	Gain coefficients of the leaky integrator (first order IIR filter) of the model	200.9E-6 200.9E-6	602.6E-6 602.6E-6

**Fig. 2:**
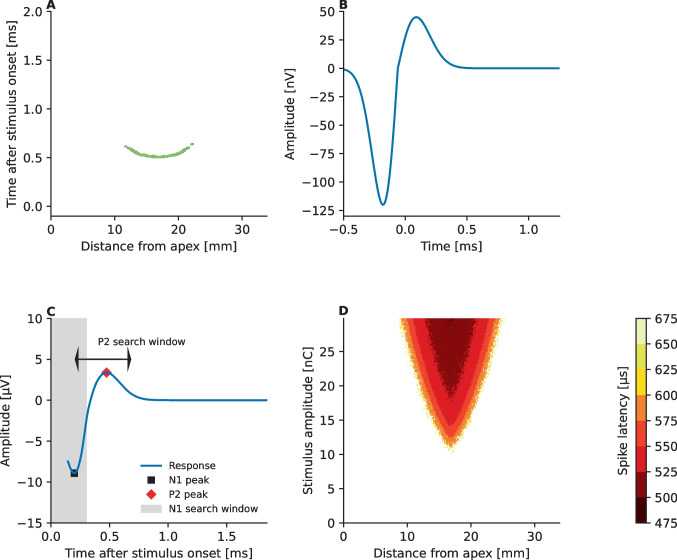
Predicted spiking activity of 500 independent ANFs distributed along the cochlear length (panel **A**) and the resulting eCAP trace (panel **C**) recorded at electrode contact #5 for a symmetric anodic-leading charge-balanced biphasic pulse (phase duration 40 µs and 2.1 µs IPG) presented at 30 nC from stimulating electrode #6. Panel **B** shows the unitary response function applied in this model. Panel **D** shows how the spiking latency of the ANFs along the simulated cochlea changes as a function of stimulus amplitude for a symmetric anodic-leading biphasic pulse (phase duration 40 µs and 2.1 µs IPG). The square and diamond symbol in panel **C** indicate the N1 and P2 maxima of the eCAP trace searched within the N1 and P2 search windows, respectively

Figure 2A depicts an example of the overall spiking activity of the modeled neurons for a symmetric anodic-leading biphasic pulse (phase duration 40 µs and IPG 2.1 µs) presented at 30 nC charge from electrode #6. The strongest and the earliest spiking activity stems from the neurons at closest proximity to the stimulating electrode. Furthermore, Fig. 2B shows how the spiking latency decreases and how increasing numbers of neurons are excited as the level of the biphasic pulse is increased incrementally from 0 to 30 nC. It should be noted that no threshold or latency differences were introduced here between anodic and cathodic polarities. Neurophysiological measurements and computational model predictions have bolstered the idea that the observed latency and threshold differences between polarities [44] could be related to degree of myelination and degeneration of the peripheral parts of the nuclei around the stimulating electrode [13, 45, 46]. Here, all ANFs were modeled to share the same parameter space of a healthy neuron, as it was deemed unnecessary to specifically adjust the degree of myelination and degeneration of the neurons at each position along the cochlea to reach the aims of this study—the effects investigated in this study are independent from such changes. Moreover, such adjustments would have required detailed etiological data to justify such parameter adjustments.

Finally, the responses of the ANFs were used to predict the electrically evoked compound action potential. To that end, spiking output of a given ANF were convolved with a physiologically realistic unitary response [47, 48] (Fig. 2B)

$$\begin{array}{c}U\left(t\right)=\left\{\begin{array}{c}\frac{{u}_{N}}{{\partial }_{N }^{2}}\left(t-{t}_{0}\right){e}^{0.5-\frac{{\left(t-{t}_{0}\right)}^{2}}{2{\partial }_{N}^{2}}}, \text{if }\:t<{t}_{0}\\ \frac{{u}_{P}}{{\partial }_{P }^{2}}\left(t-{t}_{0}\right){e}^{0.5-\frac{{\left(t-{t}_{0}\right)}^{2}}{2{\partial }_{P}^{2}}}, \text{otherwise.}\end{array}\right.\end{array}$$

Here, $t$ denotes time (ranging from − 0.6 to 1.1 ms), ${t}_{0}$ = -60 ms, ${u}_{N}$ = 12 µV, ${u}_{P}$ = 45 µV,${\partial }_{P}$ = 0.15 ms and ${\delta }_{N}$ = 0.12 ms following [47]. The eCAP response obtained at a given recording electrode was then obtained by summing up the responses from all neurons

$$V\left(t\right)=\sum_{i=1}^{N}\left({o}_{i}*U\right)\left(t\right)\times {10}^{\left(\frac{-2\left|{\overline{r} }_{r}(i)\right|}{20}\right)},$$

while considering with the same 2 dB/mm attenuation [49] of the neural response at the recording electrode. Here, $i$ denotes the index of the neuron, $N$ is the total number of modeled ANFs, ${(o}_{i}\times U)(t)$ denotes convolution between the output of the neuron (${o}_{i}$) and the unitary response, and ${\overline{r} }_{r}(i)$ is the distance vector between the given neuron and the recording electrode. Figure 2C shows the predicted eCAP response as measured at electrode #5 from the spiking activity illustrated in Fig. 2A.

### Simulations

The above model was used to simulate eCAP AGF measurements following the fine-grain stimulation paradigm [50] using alternating polarity for artifact reduction. Specifically, the stimulus amplitude was increased gradually from 0 to 30 nC with a charge-increase rate of 1.5 nC/s with the pulses being presented at a pulse rate of 80 Hz. At each stimulus amplitude, an anodic-leading and a cathodic-leading symmetric charge-balanced biphasic pulse was presented, and the responses provided by the model for the two pulses were averaged to obtain the eCAP response corresponding to the given stimulus amplitude. A reference measurement with zero amplitude stimulation, a so-called zero-amplitude template, would normally be also subtracted from the obtained eCAP response to reduce recording related artifacts independent of the stimulation, also sometimes called signature of the recording system [51, 52]. As the model did not include any initial state effects of the recording system, zero-template subtraction was omitted. The phase duration was kept fixed at 40 µs while the IPG was varied according to simulated condition. The eCAP recording window was defined to have a fixed length of 1.7 ms and to begin after a specific recording delay from the pulse onset:

$${d}_{R}=145\:\mu s+\left(i-2.1\:\mu {\text{s}}\right).$$

Here, $i$ denotes the IPG and 145 µs is the recording delay for IPG of 2.1 µs, given the phase duration of 40 µs. The eCAP amplitude corresponding to the given stimulus amplitude was then extracted by computing the amplitude difference between the positive P2 and the negative N1 peak in the obtained eCAP response (Fig. 2C), as estimated based on the local extrema within the P2 and N1 search windows. As illustrated in Fig. 2C, the negative N1 peak was searched within the first 300 µs of the eCAP recording window and the positive P2 peak was searched within a 400-µs range from the N1 peak [53].

To reach the aims of the study, simulations were performed with two IPGs (2.1 µs and 30 µs) and using the two electrode-neuron distance models described above. As mentioned previously, either 500, 1000, 1500, or 2000 ANFs were modeled to be equidistantly distributed along the cochlea to investigate the effects of neural survival on the outcome measures. It should be noted that, although the simulated 25% neural survival in the case of 500 ANFs was arbitrarily chosen to investigate the extent of the effect on the simulation results, the chosen neural survival rates reflect variation observed among CI users [54]. The electrode contact #6 in the middle of the electrode array was chosen as the stimulating electrode and eCAP recordings were simulated to be performed on all other electrode contacts (1, 2, 3, 4, 5, 7, 8, 9, 10, 11, and 12).

### Analysis

**Fig. 3:**
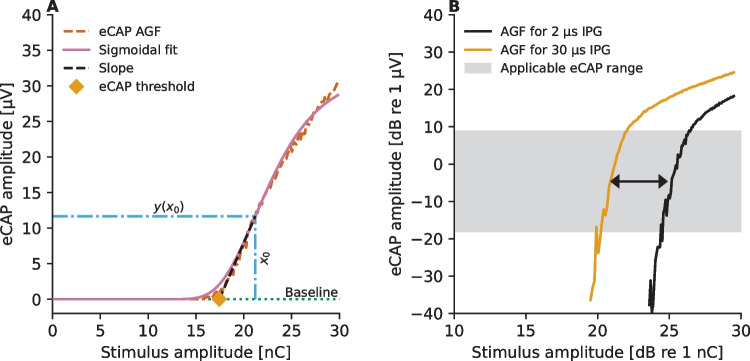
Panel **A** illustrates how the eCAP threshold and eCAP AGF slope were derived for each eCAP AGF from the parameters of a sigmoidal function that was fitted to the simulation result. Panel **B** shows how the range of the vertical axis (denoted here as “applicable eCAP range”), within which both eCAP AGFs grow linearly, was defined for calculation of the IPG offset between the two eCAP AGFs expressed in logarithmic input–output scale. The black arrow in panel **B** displays the IPG offset between the eCAP AGFs at the given eCAP amplitude

The analysis of the effects of the different parameters (IPGs, electrode-neuron distances, and neural density) on the eCAP AGFs began by fitting of a sigmoidal function

$$y(x)={y}_{0}+\frac{B}{{\left(1+{e}^{-D\left(x-C\right)}\right)}^{z}}$$

on each of the obtained stimulation results using the Levenberg–Marquardt algorithm [55]. Here, $x$ denotes the stimulus amplitude (in nC), $y(x)$ is the corresponding eCAP amplitude (in µV), ${y}_{0}$ is the baseline (the spontaneous activity of the neuron without electrical stimulation in µV), $B$ is the maximum observable eCAP amplitude at a given recording electrode (in µV), $C$ is the stimulus amplitude corresponding to the inflection point of a symmetric sigmoidal function, $D$ is the rate of change with respect to stimulation unit within the dynamic range of the neuron, and $z$ is a parameter related to the asymmetry of the sigmoid, which are the to-be-fitted parameters [56]. The asymmetry-adjusted inflection point is thus defined as ${x}_{0}=C+{\text{ln}}\left(z\right)/D$. The fitted values were then used to estimate the eCAP threshold and the eCAP AGF slope as shown in Fig. 3A: The slope, $\theta$, of the eCAP AGF at the steepest point of the sigmoid is defined by the value of the first-order derivative

$${y}{\prime}\left(x\right)=\frac{zBD{e}^{-D\left(x-C\right)}}{{\left(1+{e}^{-D\left(x-C\right)}\right)}^{z+1}} \stackrel{x=C+\frac{{\text{ln}}\left( z \right)}{D}}{\Rightarrow }BD{\left(\frac{z}{z+1}\right)}^{z+1}$$

at ${x}_{0}$ (i.e., $\theta =y{\prime}({x}_{0})$) and the stimulation intensity

$${x}_{{\text{THR}}}=C+\frac{1}{D}\left({\text{ln}}\left(z\right)-\left(\frac{z+1}{z}\right)\right),$$

at which the slope intersects with the baseline, was used to denote the eCAP threshold for a given eCAP AGF.

Subsequently, the IPG effect was computed following three alternative methods suggested in literature:

- *Absolute IPG effect*. IPG effect is defined as the difference between the slopes that are calculated in the linear input–output scale [14, 19]. Hence, the metric was calculated by simply subtracting the slope estimate obtained for 2.1 µs IPG from the one obtained for 30 µs IPG.
- *Relative IPG effect*. IPG effect is defined as the ratio between the slopes that are calculated in the linear input–output scale [12]. The metric was obtained by dividing the slope estimate obtained for 30 µs IPG by the one obtained for 2.1 µs.
- *IPG offset*. IPG effect is defined as the average offset (in dB in respect to 1 nC) between the overlapping linear portions of the eCAP AGFs obtained for the two IPGs when the corresponding eCAP AGFs are expressed in a logarithmic input–output scale [27]. For this approach the instructions given in [27] were followed: it comprised representing the eCAP AGFs in logarithmic input–output scale, manual defining the range of the vertical axis (eCAP amplitude [dB re 1 µV]) where the eCAP AGFs of both IPGs increase linearly, and computation of the average offset on the horizontal axis between them within that range. This approach was accomplished with the help of the MATLAB (Mathworks, Natick, MA) tool provided by Brochier et al. [57], which is available at https://github.com/tjbrochier/eCAP-AGF-Methods.

All IPG-effect metrics were computed separately for each combination of electrode-neuron distances and neuron densities.

## Results

**Fig. 4:**
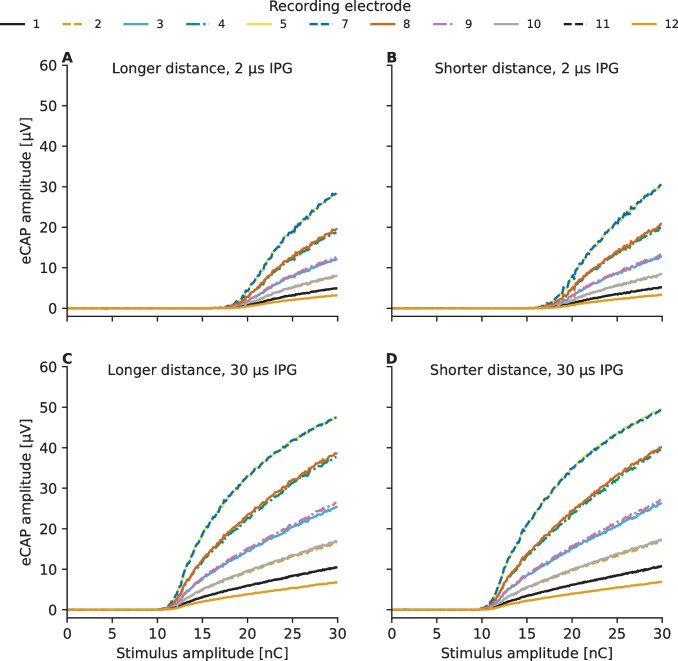
Predictions for electrically evoked compound action potential (eCAP) amplitude-growth functions (AGFs) obtained by varying the inter-phase gap (IPG) in the symmetric charge-balanced biphasic pulse (phase duration 40 µs) following fine-grain stimulation paradigm [50]. The predictions for the larger electrode-neuron distance (Fig. 1B) are shown in the left panels (panels **A** and **C**) and those for the shorter electrode-distance are shown in the right panels (panels **B** and **D**). Panels **A** and **B** show results for simulations with 2.1 µs IPG and results for 30 µs IPG are shown in panels **C** and **D**

**Fig. 5:**
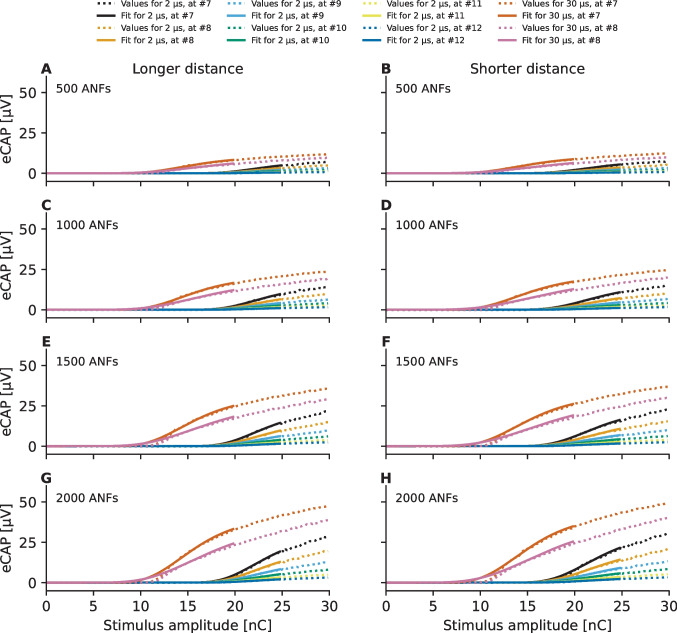
Predicted eCAP AGFs and fitted sigmoidal functions for the different conditions, with each row representing a different neural survival condition. Each panel shows the corresponding results for the six more basal electrodes (#7, #8, #9, #10, #11, and #12) and two IPGs (2.1 and 30 µs). The left and right columns display the results for the longer and shorter electrode-neuron distance, respectively. Only portions of the fitted functions and only predictions for two recording electrodes for the longer IPG are shown for visualization purposes

Figure 4 illustrates the predicted eCAP AGFs in the case of good cochlear health (2000 ANFs) for the two electrode-neuron distances (separated by columns) and the two inter-phase gap values (separated by rows) as measured at the other eleven recording electrodes (electrode contacts #1, #2, #3, #4, #5, #7, #8, #9, #10, #11, and #12). There does not seem to be any strong dependency on whether a more apical or a more basal recording electrode is used, as the predicted eCAP AGFs are similar, for instance, between recording electrodes #4 and #8 as well as between #5 and #7. Therefore, the predicted eCAP AGFs and the fitted sigmoidal functions for the different neural survival, IPG, and electrode-neuron distance conditions are shown in Fig. 5 only for the more basal recording electrodes #7, #8. #9, #10, #11, and #12.

**Fig. 6:**
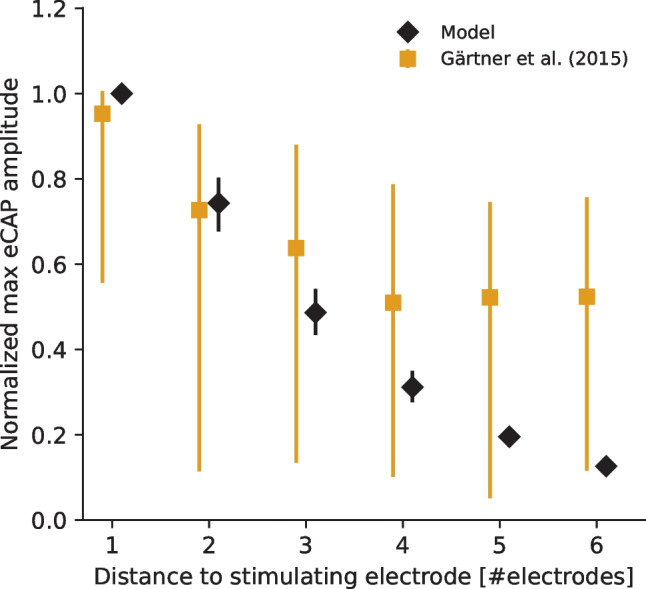
Effect of the distance between the stimulating and recording electrode on the maximum eCAP amplitude. The values have been normalized in respect to the largest value among the AGFs, separately for the different electrode-neuron distance, neural survival, and IPG condition. The values are presented along measured eCAP data from 39 ears of 35 CI users [58], with the symbols denoting the median values and the error bars representing the inter-quartile ranges

In accordance with the mathematical model formulated by Brochier et al. [27], the predicted eCAP AGFs exhibit in all cochlear health cases a distance effect related to the recording electrode as the largest eCAP amplitudes are recorded at electrode contacts (#5 and #7) that are in the closest proximity to the stimulating electrode #6. To evaluate whether the predicted decrease in maximum eCAP amplitude as a function of stimulating-recording electrode distance is in agreement with electro-physiological data from literature, the maximum eCAP amplitudes were extracted for each AGF and normalized in respect to the largest one, separately for each electrode-neuron distance, neural survival and IPG condition. The median values and the quartile ranges of the normalized values are shown in Fig. 6 along with data from 39 ears of 35 CI users [58]. The model is shown to reproduce the decrease of the maximum eCAP amplitude as a function of stimulating-recording electrode distance, with a tendency to overestimate the effect for the largest electrode distances.

**Fig. 7:**
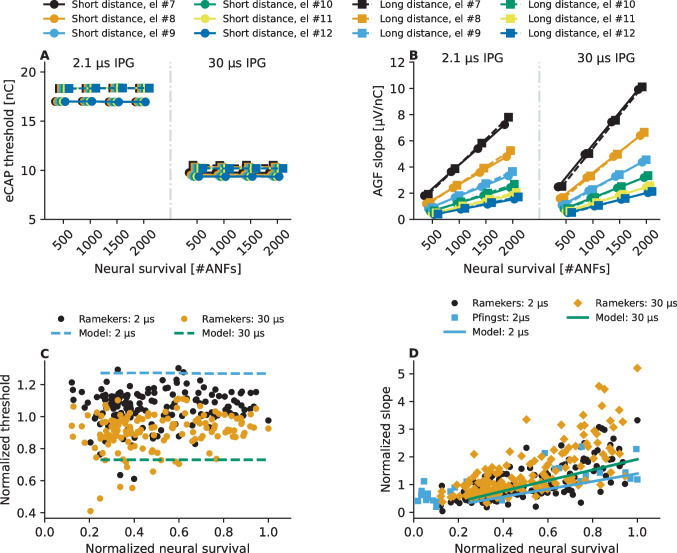
eCAP threshold (panel **A**) and eCAP AGF slope (panel **B**) estimates obtained from the simulated eCAP AGFs. The estimates were derived from parameters of a sigmoidal function that was fitted for a given eCAP AGF using the Levenberg–Marquardt [55] algorithm. Panels **C** and **D** show the normalized model predictions for the eCAP threshold and eCAP AGF slope along with the normalized neurophysiological data from literature. The datapoints labeled as "Ramekers" contain data from 154 guinea pigs measured in [14, 59, 61] and values labeled as "Pfingst" contain data from 56 guinea pigs measured in [60]

The symmetry of the predicted AGFs for basal and apical recording electrodes was reflected also in the sigmoid-fit-based eCAP threshold and eCAP AGF slope estimates and, therefore, only the values obtained for simulated recordings at the more basal electrode contacts (#7, #8, #9, #10, #11, and #12) are shown in Fig. 7. The found similarity in eCAP AGFs between an apical and a basal recording electrode is expected due to the quasi symmetric neural activation pattern around the stimulating electrode (Fig. 2A, D) and the electrode contacts being simulated to be distributed with approximately 2-mm spacing from one another following the CT data [31].

Figure 7 illustrates how the effect of the recording electrode is reflected in the slopes of the eCAP AGF becoming shallower as the distance between the stimulating and recording electrode increases, while the eCAP threshold seems to remain unaffected. Similarly, reducing the number of ANFs in the cochlear model results in shallower eCAP AGF slopes but does not affect the eCAP threshold values (Fig. 5 and Fig. 7). The opposite trend seems to be present between the two electrode-neuron distances— the eCAP thresholds being smaller for the shorter electrode-neuron distance model (Fig. 7A), while the slopes seem closely similar between the two electrode-neuron distance models. The increase in the predicted eCAP thresholds between the two electrode-neuron distance models corresponds to an average increase rate of 2.1 dB / mm. Prolonging of the IPG from 2.1 to 30 µs can be seen to result in both decreasing of the eCAP thresholds (Fig. 7A) and steepening of the eCAP AGF slope (Fig. 7B). Figures 7C and D show how the model predictions about the effects of neural survival and of prolonging IPG on the eCAP threshold and eCAP AGF characteristics match with neurophysiological data from literature [14, 59–61]. For this purpose, the values have been normalized so that the neural survival has been scaled in respect to the maximum value in the corresponding dataset and the given eCAP characteristic has been scaled in respect to the median value of the corresponding dataset.

**Fig. 8:**
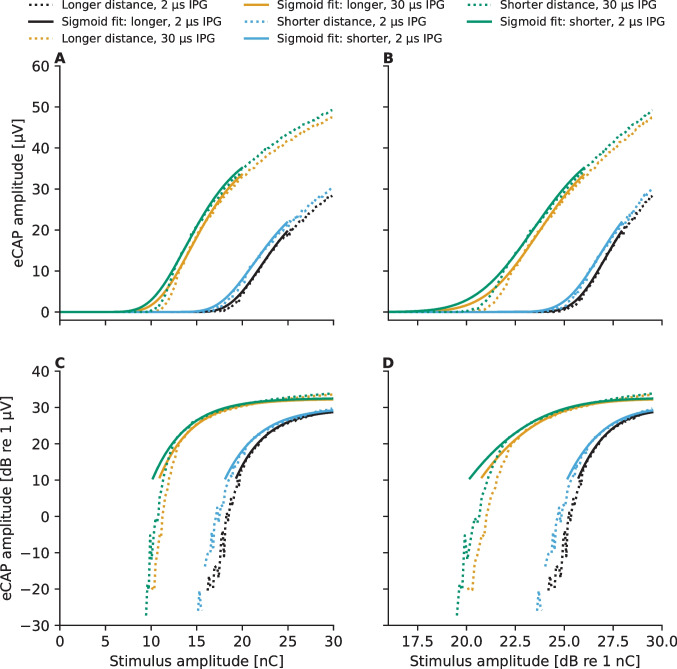
Predicted eCAP AGFs measured at electrode contact #7 for the different combinations of IPG and distance model variants in the simulations along with the fitted sigmoidal functions. The panels show the same eCAP AGFs in linear–linear (panel **A**), log-linear (panel **B**), linear-log (panel **C**), and log–log scales (panel **D**) of the stimulus amplitude and the eCAP amplitude, respectively

The mathematical model proposed by Brochier et al. [27] predicts that the effects of the non-neural contributors (electrode-neuron distance and stimulating-recording electrode distance) are eliminated from the IPG effect when the eCAP AGFs are expressed in logarithmic input–output scale. In order to test this prediction, the eCAP AGFs recorded at the electrode contact #5 (Fig. 4) were expressed in different input–output scales shown in Fig. 8. Following the prediction of the mathematical model [27], the eCAP AGFs become parallel, within the eCAP amplitude range from − 20 to 20 dB (re 1 µV), when they are expressed in the logarithmic input–output scale (Fig. 7D). The eCAP AGFs also become parallel when only the stimulus amplitude is expressed in logarithmic scale (Fig. 8B). It is also noteworthy that representation of the eCAP AGFs on a logarithmic input–output scale pronounces measurement noise, especially for small eCAP amplitudes (Fig. 8). This hinders accurate determination of the eCAP threshold.

**Fig. 9:**
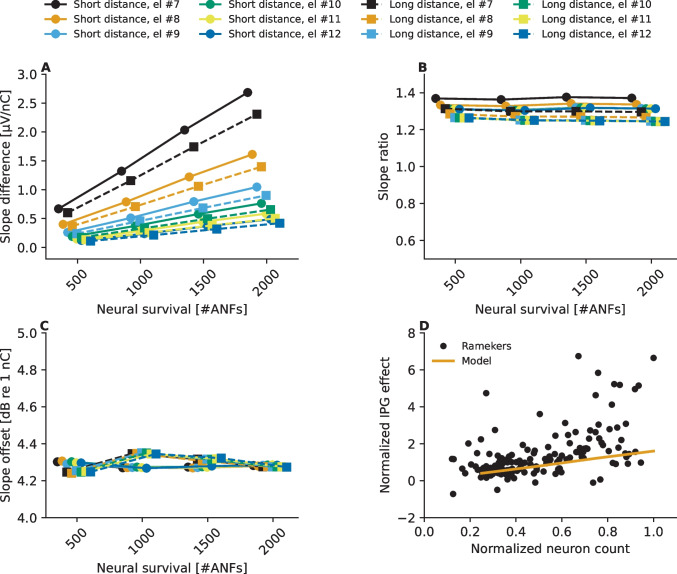
Estimated IPG effects derived from the eCAP AGFs for 2.1 and 30 µs IPG when computing the effect following either absolute IPG effect (panel **A**), the relative IPG effect (panel **B**), or the IPG offset metric (panel **C**) described in the “Methods” section. Panel **D** shows the normalized absolute IPG effect predictions along with the normalized neurophysiological data from literature. The data points labeled as "Ramekers" contain data from 154 guinea pigs measured in [14, 59, 61]

The IPG effects computed following the different methods proposed in literature are shown in Fig. 8. Interestingly, the IPG effect depends on the number of ANFs and on the stimulating-recording electrode distance but only when the IPG effect is computed following the *absolute IPG effect* method as the difference between the eCAP AGF slopes expressed in the linear input–output range (Fig. 9A). Predicted IPG effects computed following either the *relative IPG effect* (Fig. 9B) or the *IPG offset* methods (Fig. 3B, C) do not show clear dependency on any of the evaluated factors.

## Discussion

The present study employed a computational model to investigate how the IPG effect-based metrics of cochlear health are affected by neural survival and non-neural aspects of the electrode-neuron interface inside the cochlea. The reported evidence about the IPG effect correlating with cochlear health in non-human mammals [12, 14, 19] has been questioned due to the difficulties in translating eCAP-based metrics into clinical practice with CI users [23, 24, 62]. Moreover, there is debate about to what extent such non-invasive measurements of cochlear health are influenced by non-neural aspects such as the electrode-neuron distance [15, 16, 26]. It has been further questioned how the slope of the eCAP AGF should be estimated [63] and how the IPG effect should be computed from the eCAP AGFs in order to minimize the effects of non-neural aspects on the results [27, 57].

The model presented in this work was designed to simulate how the electrical current spreads in a simplified 2-D model of an implanted cochlea, and how the ANFs distributed along the cochlea respond to the electrical stimulus reaching them, resulting in action potentials that are then recorded at specific electrode contact(s) to measure the evoked eCAP responses. The electrode locations of a lateral wall electrode array were derived from post-operative CT scans [31]. The ANFs were assumed to be independent and equidistantly distributed along the cochlear duct. Scala tympani height data [34] was used to approximate the distance from the electrode array to the ANFs at different positions along the cochlea. Both the electrical stimulus reaching the individual ANF as well the ANF’s response reaching the recording electrode were assumed to be attenuated by 2 dB/mm [49] Hence, each ANF received the electrical stimulus attenuated depending on the corresponding Euclidean distance from the stimulating electrode. The ANF responses were obtained by using a phenomenological model [40] to predict the spiking activity of the given neuron and by convolving the spikes with a unitary-response function [47]. The eCAP response was then constructed by combining the attenuated responses of the individual neurons, where the attenuation depended on the Euclidean distance from the given neuron to the recording electrode. The total number of ANFs (500, 1000, 1500, and 2000) was varied to evaluate the effects of neural density. Two IPGs (2.1 and 30 µs) and two electrode-neuron distances (i.e., two scala tympani height vectors), as well as eleven recording electrodes, were selected for testing the effects of the parameter combinations on eCAP AGF measurements performed following the fine-grain stimulation paradigm [50] using alternating polarity for artifact reduction.

The model predictions showed that prolonging of the IPG resulted in both decreased eCAP thresholds and steeper eCAP AGF slopes. Both outcomes are in accordance with existing literature: Neurophysiological measurements with biphasic pulses [1] have demonstrated that the addition of an IPG between the two polarities allows a lower intensity pulse to excite the ANF as the leading polarity has more time to evoke an action potential before the second polarity abolishes the spiking activity. Also, in the case of anodic-sensitive site of excitation being stimulated with cathodic-leading pulse, or vice versa, IPG can be thought to provide the cell membrane with more time to recover from the hyperpolarization caused by the preceding non-excitatory polarity. Therefore, the increase of eCAP response amplitude with stimulus amplitude is pronounced in the case of longer IPG because the longer IPG increases the likelihood of ever larger population of neurons being excited at a given stimulus amplitude. The reason why prolonging of the IPG is more pronounced in healthy cochleae [14] could be related to the critical period [64]— the time within which the action potential initialization process must be completed before the second polarity abolishes the spiking activity —being shorter in demyelinated fibers and/or to there being less neurons available to be recruited due to degeneration of the fibers [14, 65, 66].

The number of ANFs in the model affected the eCAP AGF slope but had no effect on the predicted eCAP thresholds. In agreement with this model prediction, no significant effects of neural density on eCAP threshold values or on psychophysical detection thresholds for single pulses of implanted guinea pigs have been found in previous studies [14, 19, 59, 61] (Fig. 7C).

Two kinds of distance effects were observed in the model predictions. On one hand, only the eCAP threshold was affected by the electrode-neuron distance—lower threshold being predicted for the shorter electrode-neuron distance. On the other hand, increase in the stimulating-recording electrode distance was found to result in shallower eCAP AGF slopes, while the eCAP threshold remained the same. The observed dependency of the eCAP threshold on the electrode-neuron distance is in agreement with Schwartz-Leyzac et al. [26], although the predicted 2.1 dB/mm effect is larger than what that study reported based on eCAP and post-operative CT data of CI users. Here, the effect is related to the manner by which the spread of electrical field was considered in the model. Due to the 2 dB/mm attenuation, a pulse with a given amplitude builds up less membrane potential when the electrode-neuron distance is larger. As the membrane voltage and the threshold potential of the neuron fluctuate over time [67, 68], the increased electrode-neuron distance reduces the likelihood of a given neuron to release an action potential. Phenomenological models such as the one applied here [40] capture this by using a cumulative distribution function of a normal distribution to map the electrical current to spiking probability [69]. Here, the slopes of the eCAP AGF are not strongly affected because the effect is highly similar across different electrode-neuron combinations between the two electrode-neuron distance conditions (Fig. 1B). In contrast, the stimulating-recording electrode distance affects the slope of the eCAP AGF and the maximum eCAP amplitude but not the eCAP threshold because mostly the neurons in the proximity of the stimulating electrode are excited (Fig. 2B) and, therefore, the eCAP amplitudes decrease with increased stimulating-recording electrode distance.

The predicted IPG effect on the eCAP AGF depended not only on the neural survival but also on how the IPG effect was calculated. Only when the *absolute IPG effect* was calculated as the difference between the slopes on a linear input–output scale, did the IPG effect depend on the neural survival— bigger effects being predicted to be obtained in the case of better ANF survival —as found in neurophysiological studies with non-human mammals [14, 19]. The *absolute IPG effect* was found to be influenced also by non-neural aspects, as hypothesized by Brochier et al. [27], with the predicted effect decreasing when either the stimulating-recording electrode distance or the electrode-neuron distance was increased. However, eCAPs are for these very reasons nominally measured at recording electrodes in the vicinity of the stimulating electrode (i.e., ± two electrode contacts) and the same recording electrode should be used to measure responses for both IPGs. Within such a range, effects of the non-neural aspects were found to be marginal here compared to the impact of neural survival on the *absolute IPG effect*. When either the *relative IPG effect* (ratio between the slopes estimated on linear input–output scale) or the *IPG offset* [27] was calculated, influences of non-neural aspects disappeared, following the mathematical model and the reasoning presented by Brochier et al. [27]. However, the dependency of the IPG effect on the neural survival was also not evident when either the *relative IPG effect* or the *IPG offset* was used.

The finding that computation of the *IPG offset* also removes the influence of the neural survival rate from the results is in line with the predictions obtained by Brochier et al. [57] based on their computational model. In [57], the authors used the phenomenological model from Joshi et al. [70] and investigated the dependency of the model’s spiking rate for time-invariant pulse trains on stimulus amplitude and number as well as the neural health of the modeled neurons. Their results showed that only the properties of the neuron to affect the predicted *IPG offset*, based on which Brochier et al. [57] suggested that the *IPG offset* could be a measure of the demyelination of the central parts of the ANF, especially when the peripheral parts have been degenerated. For practical applications, the *IPG offset* suffers from the disadvantage that representation of the eCAP AGFs on a logarithmic input–output scale biases the impact of the noise on the AGF as illustrated in Fig. 8. However, this could be overcome by restricting measurements to a range where signal-to-noise ratios are high and/or applying a higher weight at larger SNRs.

The present study did not investigate effects of gradual degeneration of the ANFs. Therefore, this approach does not allow evaluating if either the *relative IPG effect* or the *IPG offset* would be more indicative of the gradual degeneration of the neurons than the *absolute IPG effect*, as hypothesized by Brochier et al. [27]. The topic of dead regions was also not addressed here, as only the density of the neurons along the cochlea was varied. It is likely that in the effect of stimulating-recording electrode distance would change if the neurons in the proximity of the stimulating electrode would be simulated as being degenerated. As stated before (see Methods), the fidelity of the 2-D model could be refined in several ways, such as by using more natural distribution of the ANFs along the cochlea [35], considering the orientation of the electrodes and/or asymmetric spread of the electrical field [38], and/or including threshold and latency differences between the polarities [44]. Although the lack of such fidelity refinements does not affect the validity of the observations in the present study, and some refinements would themselves likely introduce biases, they offer an interesting topic for future work. Another interesting topic for future work would comprise simulating eCAP recordings with different types of electrode arrays and/or stimulation modes, as they could change the contributions of the non-neural aspects on the results [71]. The present framework enables inclusion of such improvements.

One of the unanswered questions in the field is which aspect of the cochlear health is of importance considering the hearing outcomes of the CI user. Neurophysiological studies on non-human mammals have shown how aspects of neural health and neural survival are reflected in objective measures. The present study and the works by Brochier et al. [27, 57] have demonstrated how computational models can be used to predict how objective metrics, such as the IPG effect, of cochlear health depend not only on the neural aspects, but as well as on non-neural aspects and how the metric is computed. Increased knowledge of such dependencies may help future clinical applications and investigators to accommodate the heterogeneity among CI users (their etiologies, durations of deafness, age at implantation, etc.) and to develop better correlations between cochlear-health metrics and hearing outcomes.

## Data Availability

The original MATLAB (MathWorks, Natick, MA) code of the phenomenological single-fiber model used in this study is available at 10.5281/zenodo.4674563.
